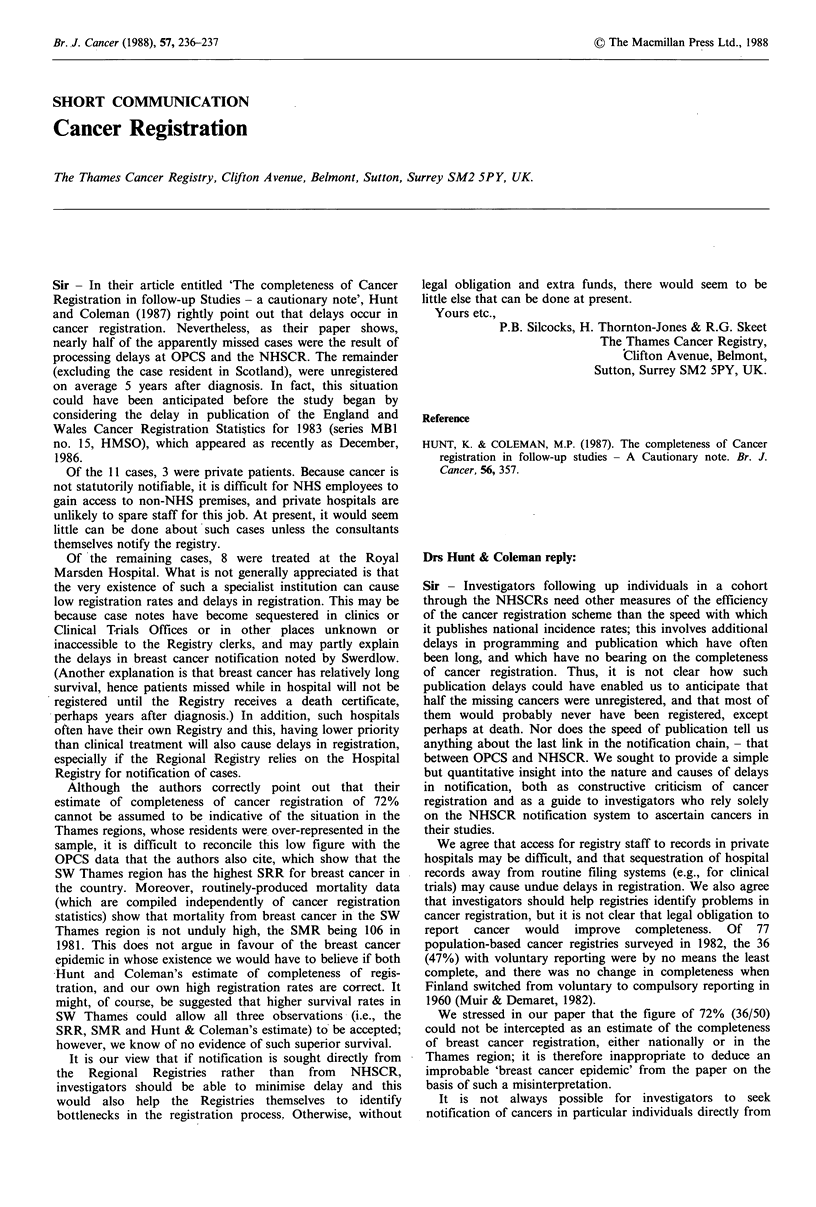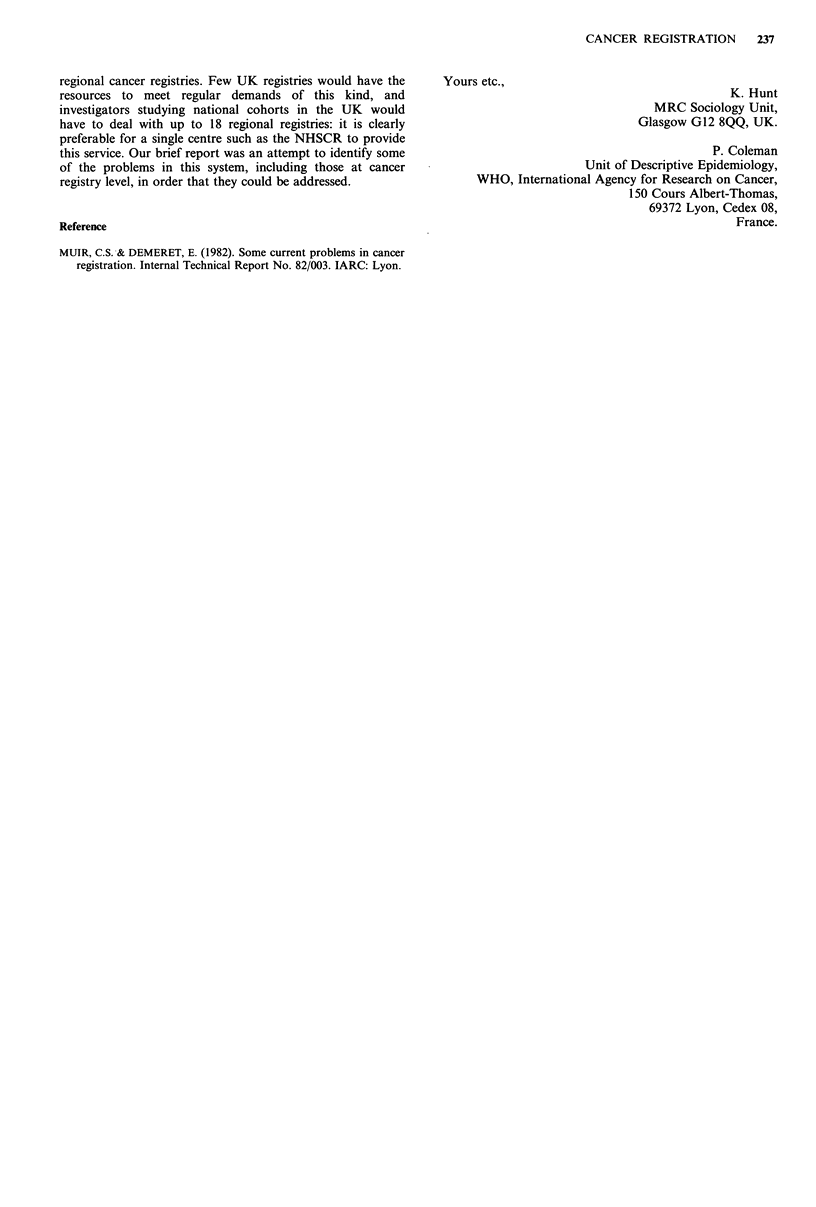# Drs Hunt & Coleman reply

**Published:** 1988-02

**Authors:** K. Hunt, P. Coleman


**Reference**

HUNT, K. & COLEMAN, M.P. (1987). The completeness of Cancer registration in follow-up studies – A Cautionary note. *Br. J. Cancer*, **56**, 357.

**Drs Hunt & Coleman reply:**

**Sir** – Investigators following up individuals in a cohort through the NHSCRs need other measures of the efficiency of the cancer registration scheme than the speed with which it publishes national incidence rates; this involves additional delays in programming and publication which have often been long, and which have no bearing on the completeness of cancer registration. Thus, it is not clear how such publication delays could have enabled us to anticipate that half the missing cancers were unregistered, and that most of them would probably never have been registered, except perhaps at death. Nor does the speed of publication tell us anything about the last link in the notification chain, – that between OPCS and NHSCR. We sought to provide a simple but quantitative insight into the nature and causes of delays in notification, both as constructive criticism of cancer registration and as a guide to investigators who rely solely on the NHSCR notification system to ascertain cancers in their studies.

We agree that access for registry staff to records in private hospitals may be difficult, and that sequestration of hospital records away from routine filing systems (e.g., for clinical trials) may cause undue delays in registration. We also agree that investigators should help registries identify problems in cancer registration, but it is not clear that legal obligation to report cancer would improve completeness. Of 77 population-based cancer registries surveyed in 1982, the 36 (47%) with voluntary reporting were by no means the least complete, and there was no change in completeness when Finland switched from voluntary to compulsory reporting in 1960 (Muir & Demaret, 1982).

We stressed in our paper that the figure of 72% (36/50) could not be intercepted as an estimate of the completeness of breast cancer registration, either nationally or in the Thames region; it is therefore inappropriate to deduce an improbable 'breast cancer epidemic' from the paper on the basis of such a misinterpretation.

It is not always possible for investigators to seek notification of cancers in particular individuals directly from regional cancer registries. Few UK registries would have the resources to meet regular demands of this kind, and investigators studying national cohorts in the UK would have to deal with up to 18 regional registries: it is clearly preferable for a single centre such as the NHSCR to provide this service. Our brief report was an attempt to identify some of the problems in this system, including those at cancer registry level, in order that they could be addressed.

**Reference**

MUIR, C.S. & DEMERET, E. (1982). Some current problems in cancer registration. Internal Technical Report No. 82/003. IARC: Lyon.

Yours etc.,

K. Hunt
MRC Sociology Unit,
Glasgow G12 8QQ, UK.

P. Coleman
Unit of Descriptive Epidemiology,
WHO, International Agency for Research on Cancer,
150 Cours Albert-Thomas,
69372 Lyon, Cedex 08,
France.